# Supplementary material for: Self-guided versus facilitator-guided debriefing in immersive virtual reality simulation: Protocol for a randomized controlled non-inferiority trial assessing teamwork skills in medical students
Source: PLoS One. 2025 Sep 12;20(9):e0332309. doi: 10.1371/journal.pone.0332309 (PMC12431211; doi:10.1371/journal.pone.0332309)
Supplement: S2 File — (PDF) [file pone.0332309.s002.pdf]

Projektets titel: Tryg i læring: Forbedret modtagelse og behandling af akut syge børn gennem træning i Virtual Reality

Forsøgsansvarlig:
Amalie Middelboe Andersen, MD.
Ansat i forskningsstilling på Afdeling for Børn og Unge, Juliane Marie Centret, Rigshospitalet.
amalie.middelboe.andersen@regionh.dk
tlf. 25 34 46 10

Vejledere:
Jesper Kjærgaard, MD, Ph.d., Afdeling for Børn og Unge, Rigshospitalet
Jette Led Sørensen, MD, Ph.d., MEEd, Professor i Interprofessionel Læring, Mary Elizabeth's Hospital og Juliane Marie Centeret, Rigshospitalet
Anja Poulsen, MD, Ph.d., Afdeling for Børn og Unge, Rigshospitalet

Projektets baggrund

De akutte og livstruende tilstande hos børn er heldigvis sjældne, men netop derfor får sundhedsprofessionelle ikke opøvet tilstrækkelig rutine gennem det daglige arbejde. Derfor trænes håndteringen af kritisk syge børn gennem simulerede scenarier. Dette foregår oftest ved at et team af sundhedsprofessionelle samles omkring en simulationsdukke, og en uddannet facilitator guider teamet igennem et simuleret scenarie og efterfølgende faciliterer læring og refleksion gennem debriefing. Træningen er dog ressourcekrævende og afhængig af høj uddannelsesmæssig ekspertise i den enkelte afdeling.

Virtual Reality (VR) er en teknologi der har vundet stort indpas til simulation i sundhedsvæsenet. Her kan laves computerprogrammerede træningsscenarier, der kan skaleres på tværs af afdelinger og regioner. VR-simulation har vist stor effekt til træning af sundhedsprofessionelle i en lang række specialer, men der mangler studier, der undersøger, om VR er effektivt til at træne sundhedsprofessionelle teams i håndtering af det akut syge barn, samt studier der undersøger, hvordan debriefing bedst tilrettelægges i teambaseret VR.

Projektets formål

Det overordnede formål med projektet er at øge kvaliteten af undervisningstilbud til

sundhedsprofessionelle og studerende på børneafdelingerne på Rigshospitalet og Odense Universitetshospital.

Projektets metode

Studie 1: Udvikling af et framework for debriefing i VR-baseret akut pædiatri simulation.

I dette kvalitative studie vil nationale og internationale eksperter i simulationsbaseret læring blive inviteret til at afprøve VR-simulationen. Efterfølgende vil der blive afholdt individuelle, semi-strukturerede interviews samt fokusgrupper mhp. at identificere facilitatorer og barrierer for debriefing i VR-simulationen. Interviews med nationale eksperter vil foregå på Afdeling for Børn og Unge, Rigshospitalet. Interviews med internationale eksperter vil foregå online via Microsoft Teams 365.

De kvalitative data vil blive analyseret, og på baggrund heraf vil der blive udviklet en guide til sundhedsprofessionelle der skal facilitere debriefing i teambaseret VR-simulation.

Studie 2: Pilottestning af et debriefing framework i VR-baseret akut pædiatri simulation.

I dette pretest-posttest randomiserede studie vil læger og sygeplejersker fra børneafdelingerne på Rigshospitalet og Odense Universitetshospital blive randomiseret til team-baseret VR-simulation med debriefing (intervention) og uden debriefing (kontrol.) Begge grupper vil før og efter VR-simulationen blive filmet i et simuleret scenarie med simulationsdukke. En bedømmer blændet for interventionsstatus vil gennemse videoerne og vurdere personalets færdigheder ud fra en valideret checkliste.

Det primære endemål er deltagernes færdigheder bedømt ud fra den validerede checkliste.

Ud fra spørgeskemaer vil der blive indsamlet demografiske data, data på deltagernes tilfredshed med debriefingen, og deltagernes subjektive opfattelse af psykologisk tryghed i teamet. Endvidere vil der ud fra et valideret multiple choice questionnaire blive opsamlet data på deltagernes situationsbevidsthed i scenariet.

Dataindsamlingen vil foregå på Afdeling for Børn og Unge, Rigshospitalet samt på H.C. Andersens Børne- og Ungehospital, Odense Universitetshospital.

Studie 3: Self-guided debriefing vs facilitator-guided debriefing i VR-baseret akut pædiatri simulation.

I dette randomiserede, kontrollerede studie, vil medicinstuderende, læger og sygeplejersker blive randomiseret til team-baseret VR-simulation med *facilitator-guided* debriefing (kontrol) eller VR-simulation med *self-guided* debriefing (intervention).

Før og efter VR-simulationen vil begge grupper blive filmet i et simuleret scenarie med simulationsdukke.

En bedømmer blændet for interventionsstatus vil gennemse videoerne og vurdere personalets færdigheder ud fra en valideret checkliste.

Det primære endemål er deltagernes færdigheder bedømt ud fra den validerede checkliste.

Ud fra spørgeskemaer vil der blive indsamlet demografiske data, data på deltagernes tilfredshed med debriefingen, og deltagernes subjektive opfattelse af psykologisk tryghed i teamet. Ud fra et valideret multiple choice questionnaire vil der endvidere blive opsamlet data på deltagernes situationsbevidsthed i scenariet.
Dataindsamlingen vil foregå på Afdeling for Børn og Unge, Rigshospitalet samt på H.C. Andersens Børne- og Ungehospital, Odense Universitetshospital.
Protokollen for studiet planlægges udgivet.

Deltagere

Studie 1: Deltagerne vil bestå af nationale og internationale eksperter indenfor VR baseret læring og simulation (læger, psykologer og software udviklere med relevant ekspertise). Ca. 10 eksperter vil indgå i studiet.
Ovenfor nævnte eksperter vil blive kontaktet via email med skriftlig deltagerinformation. Ved interesse for deltagelse vil der yderligere gives mundtligt deltagerinformation og sendes samtykkeerklæring. Inden påbegyndelse af interviews vil deltagere underskrive samtykkeerklæring.
Ingen patienter eller biologisk materiale vil indgå i studiet.

Studie 2 og 3: Deltagerne vil bestå af læger og sygeplejersker ansat på Afdeling for Børn og Unge, Rigshospitalet, og H.C. Andersen Børne- og Ungehospital, Odense Universitetshospital, samt medicinstuderende fra Region Hovedstaden, Region Sjælland og Region Syd. I studie 2 vil indgå ca. 10 læger og 20 sygeplejersker (convenience sample). I studie 3 vil indgå ca. 90 medicinstuderende, baseret på powerberegning foretaget med G*Power© software med power sat til 80% og α=0.05.
Sundhedsprofessionelle og studerende med interesse i deltagelse vil modtage mundtlig og skriftlig deltagerinformation og samtykkeerklæring. Før aktiviteter relateret til forsøgsdeltagelse, vil deltagere underskrive samtykkeerklæring.
Ingen patienter eller biologisk materiale vil indgå i forsøget.

Hvad der måles på

I studie 1 indsamles kvalitative data fra eksperter i simulationsbaseret læring (læger, psykologer og software-udviklere med relevant ekspertise). Denne data indsamles ved at optage interviews på diktafon med talegenkendelse samt gennem noter skrevet under interviews. Alt elektronisk data overføres efter indsamling til et L-drev, mens fysisk materiale vil være anonymt (uden navne på) og opbevares på

Rigshospitalet, Afsnit 94A-2-2, i et låst arkivskab i et aflåst rum (277). Ved analyse vil vi trække anonymiserede data ud, som opbevares på L-drevet.

I studie 2 og 3 vil spørgeskemaerne distribueres via REDCap (Research Electronic Data Capture), hvor deltagerne giver samtykke til deltagelse ved at aktivere spørgeskemalinket. Ved analyse vil vi trække anonymiserede data ud, som opbevares på L-drevet. Videooptagelserne vil blive overført fra lokal lagring på videokamera til et L-drev. Videoerne vil blive slettet efter endt databehandling. Ved analyse vil vi trække anonymiserede data ud, som opbevares på L-drevet.
Multiple choice questionnaires vil blive udfyldt anonymt (uden navne på) og opbevaret på Rigshospitalet, Afsnit 94A-2-2 i et låst arkivskab i et aflåst rum (277).
Vi vil anvende Excel (version 2016, Microsoft Corp., Redmond, WA, USA) og R (version 4.0.1, R Foundation for Statistical Computing, Vienna, Austria) til at analysere data.
