# Supplementary material for: Self-guided versus facilitator-guided debriefing in immersive virtual reality simulation: Protocol for a randomized controlled non-inferiority trial assessing teamwork skills in medical students
Source: PLoS One. 2025 Sep 12;20(9):e0332309. doi: 10.1371/journal.pone.0332309 (PMC12431211; doi:10.1371/journal.pone.0332309)
Supplement: S3 File — (PDF) [file pone.0332309.s003.pdf]

**Project title:** Safe learning environment: Improved management and treatment of acutely ill children through training in virtual reality

**Principal investigator**:
Amalie Middelboe Andersen, MD.
Employed in a research position at the Department of Pediatrics and Adolescent Medicine, Copenhagen University Hospital – Rigshospitalet
amalie.middelboe.andersen@regionh.dk
+45 25 34 46 10

**Supervisors:**
Jesper Kjærgaard, MD, Ph.D. Department of Pediatrics and Adolescent Medicine, Copenhagen University Hospital – Rigshospitalet
Jette Led Sørensen, MD, Ph.D., MEEd, Professor in Interprofessional Learing, Mary Elizabeth's Hospital and the Juliane Marie Centre, Copenhagen University Hospital – Rigshospitalet
Anja Poulsen, MD, Ph.D., Department of Pediatrics and Adolescent Medicine, Copenhagen University Hospital – Rigshospitalet

**Project background**

Fortunately, acute and life-threatening conditions in children are rare, but precisely because of this, healthcare professionals do not gain sufficient routine through their daily work. Therefore, the management of critically ill children is trained through simulated scenarios. This usually takes place by gathering a team of healthcare professionals around a mannequin, and a trained facilitator guides the team through a simulated scenario and subsequently facilitates learning and reflection through debriefing. However, the training is resource-intensive and depends on a high level of educational expertise in the individual department.

Virtual Reality (VR) is a technology that has gained widespread traction for simulation in healthcare. It allows the development of computer-programmed training scenarios that can be scaled across departments and regions.

VR simulation has proven effective in training healthcare professionals in a wide range of specialties, but there is a lack of studies investigating whether VR is effective in training healthcare teams in handling critically ill children, as well as studies investigating how debriefing can best be organized in team-based VR.

**The purpose of the project**

The overall aim of the project is to improve the quality of training offered to healthcare professionals and students in the pediatric departments at Rigshospitalet and Odense University Hospital.

**Project method**

*Study 1: Development of a framework for debriefing in VR-based pediatric emergency simulation.*

In this qualitative study, national and international experts in simulation-based learning will be invited to test the VR simulation. Subsequently, individual, semi-structured interviews and focus groups will be held to identify facilitators and barriers to debriefing in VR simulation. Interviews with national experts will take place at the Department of Children and Adolescent Medicine, Rigshospitalet. Interviews with international experts will take place online via Microsoft Teams 365.

The qualitative data will be analyzed, and based on this, a debriefing script will be developed for healthcare professionals who will facilitate debriefing in team-based VR simulation.

*Study 2: Pilot testing of the debriefing framework in VR-based pediatric emergency simulation.*

In this pretest-posttest randomized study, doctors and nurses from the pediatric departments at Rigshospitalet and Odense University Hospital will be randomized to team-based VR simulation with debriefing (intervention) and without debriefing (control). Before and after the VR simulation, both groups will be filmed in a simulated scenario with a mannequin. An assessor blinded to the intervention status will review the videos and assess the staff's skills based on a validated checklist.

The primary endpoint is the participants' skills assessed based on the validated checklist.

Questionnaires will be used to collect demographic data, data on the participants' satisfaction with the debriefing, and the participants' subjective perception of psychological safety in the team. Furthermore, a validated multiple-choice questionnaire will be used to collect data on the participants' situational awareness in the scenario.

Data collection will take place at the Department of Children and Adolescent Medicine, Rigshospitalet, and at H.C. Andersen's Children's Hospital, Odense University Hospital.

*Study 3: Self-guided debriefing vs. facilitator-guided debriefing in VR-based pediatric emergency simulation.*

In this randomized, controlled study, medical students, doctors, and nurses will be randomized to team-based VR simulation with *facilitator-guided* debriefing (control) or VR simulation with *self-guided* debriefing (intervention).

Before and after the VR simulation, both groups will be filmed in a simulated scenario with a mannequin. An assessor blinded to the intervention status will review the videos and assess the staff's skills based on a validated checklist.

The primary endpoint is the participants' skills assessed based on the validated checklist.

Questionnaires will be used to collect demographic data, data on participants' satisfaction with the debriefing, and participants' subjective perception of psychological safety in the team. A validated multiple-choice questionnaire will also be used to collect data on participants' situational awareness in the scenario.

Data collection will take place at the Department of Children and Adolescent Medicine, Rigshospitalet, and at H.C. Andersen's Children's Hospital, Odense University Hospital.

The study protocol is planned to be published.

**Participants**

Study 1: Participants will consist of national and international experts in VR-based learning and simulation (doctors, psychologists, and software developers with relevant expertise). Approximately 10 experts will participate in the study.

The above-mentioned experts will be contacted by email with written participant information. If interested in participating, further verbal participant information will be provided, and a consent form will be sent. Before the interviews begin, participants will sign a consent form.

No patients or biological material will be included in the study.

Studies 2 and 3: Participants will consist of doctors and nurses employed at the Department of Children and Adolescent Medicine, Rigshospitalet, and H.C. Andersen Children's Hospital, Odense University Hospital, as well as medical students from the Capital Region of Denmark, Region Zealand, and the Region of Southern Denmark. Study 2 will include approximately 10 doctors and 20 nurses (convenience sample). Study 3 will include approximately 90 medical students, based on a power calculation performed with G*Power© software with power set to 80% and $\alpha=0.05$.

Healthcare professionals and students interested in participating will receive verbal and written participant information and a consent form. Before activities related to trial participation, participants will sign a consent form.
No patients or biological material will be included in the trial.

**What will be measured**
In study 1, qualitative data will be collected from experts in simulation-based learning (doctors, psychologists, and software developers with relevant expertise). This data will be collected by recording interviews on a voice recorder with speech recognition and through notes taken during interviews. All electronic data will be transferred to a secure hospital drive as logged files after collection, while physical material will be anonymized (without names) and stored at Rigshospitalet, Section 94A-2-2, in a locked filing cabinet in a locked room (277). For analysis, we will extract anonymized data stored on a secure hospital drive as logged files.

In studies 2 and 3, the questionnaires will be distributed via REDCap (Research Electronic Data Capture), where participants give their consent to participate by activating the questionnaire link. During analysis, we will extract anonymized data, which will be stored on a secure hospital drive as logged files. The video recordings will be transferred from local storage on the video camera to a secure hospital drive as logged files. The videos will be deleted after data processing is complete. During analysis, we will extract anonymized data, which will be stored on a secure hospital drive as logged files. Multiple choice questionnaires will be completed anonymously (without names) and stored at Rigshospitalet, Section 94A-2-2 in a locked filing cabinet in a locked room (277).
We will use Excel (version 2016, Microsoft Corp., Redmond, WA, USA) and R (version 4.0.1, R Foundation for Statistical Computing, Vienna, Austria) to analyze the data.
