# Supplementary material for: Self-guided versus facilitator-guided debriefing in immersive virtual reality simulation: Protocol for a randomized controlled non-inferiority trial assessing teamwork skills in medical students
Source: PLoS One. 2025 Sep 12;20(9):e0332309. doi: 10.1371/journal.pone.0332309 (PMC12431211; doi:10.1371/journal.pone.0332309)
Supplement: Attachment — Submitted filename: complete peer review of PONE-D-25-26268.pdf [file pone.0332309.s004.pdf]

Here is a complete peer review of the manuscript titled **"Self-guided versus facilitator-guided debriefing in immersive virtual reality simulation: Protocol for a randomized controlled non-inferiority trial assessing teamwork skills in medical students"** (PLOS ONE: Review assignment for PONE-D-25-26268 is due soon - [EMID:80d903e4cc9d8459]), based on the PLOS ONE review form:

Recommendation: Minor Revisions

1. Does the manuscript provide a valid rationale for the proposed study, with clearly identified and justified research questions?

Yes

Comments:
The manuscript provides a well-articulated rationale grounded in relevant literature for evaluating self-guided versus facilitator-guided debriefing in immersive VR-based simulation. The identified research gap is significant, given the increasing adoption of VR in medical education and the need to assess the pedagogical equivalence of debriefing methods. The research questions are clearly stated, aligned with the study objectives, and justified based on the results of a preceding pilot study.

2. Is the protocol technically sound and planned in a manner that will lead to a meaningful outcome and allow testing the stated hypotheses?

Yes

Comments:
The study is designed as a randomized, controlled, non-inferiority trial with appropriate blinding of raters and a robust pretest-posttest structure. The use of validated assessment tools and statistical models (e.g., constrained linear mixed models) supports the technical rigor of the protocol. The inclusion of a power analysis based on pilot data adds strength to the proposed sample size. Exploratory outcomes, assumptions, and blinding are adequately addressed.

3. Is the methodology feasible and described in sufficient detail to allow the work to be replicable?

Yes but

Comments:
Strengths:

- The study is methodologically rigorous and adheres to the SPIRIT guidelines.
- It employs a well-defined randomized controlled, single-blinded, non-inferiority design, which is appropriate to answer the stated research questions.
- The use of validated and standardized assessment tools (e.g., CTS, ABCDE checklist, DASH-SV, IMI, NASA-TLX, SUS, VRSQ) strengthens the internal validity and reproducibility.
- Clear blinding procedures for outcome raters and detailed randomization methodology are commendable.
- The intervention and assessment phases are well-illustrated with timelines and scenarios.
- Considerations for VR-induced side effects (cybersickness) and ethical approvals are properly addressed.

Comments for Improvement:

1. **Clarify rationale for non-inferiority margin:**
    - The chosen margin of 0.5 on the CTS scale appears derived from pilot data, but a clearer justification of its clinical relevance is warranted. The margin should reflect what constitutes a non-meaningful difference in teamwork skills.
2. **Stratified randomization detail:**
    - Although stratified randomization is mentioned, the strata used (e.g., gender, prior VR/simulation experience) are not clearly specified. This should be detailed to ensure group comparability.
3. **Team composition and matching:**
    - The formation of teams (pairs of students) may introduce variability. Consider clarifying whether any matching criteria (e.g., prior teamwork experience or clinical rotation level) were used to reduce heterogeneity across teams.

4. **Handling missing data:**
    - The statistical plan notes an intention to account for missing data using mixed models, but further detail on handling attrition, particularly in individual-level data (e.g., imputation or exclusion), should be provided.
5. **Blinding limitations:**
    - While rater blinding is described well, the lack of participant blinding may introduce performance bias. Although understandable, it could be useful to acknowledge this limitation more explicitly and discuss mitigation strategies.
6. **Assessment of carryover effect:**
    - As the same individuals are involved in both pre- and post-tests with different scenarios, consideration of potential carryover learning effect or learning due to exposure (rather than intervention) is warranted.
7. **Data safety and monitoring:**
    - The manuscript would benefit from a brief mention of any data monitoring committee or oversight mechanism, particularly in the context of student participants and recorded video data.

4. Have the authors described where all data underlying the findings will be made available when the study is complete?

Yes

Comments:
The authors have declared that no datasets were generated at this stage and that deidentified data will be made available upon completion of the study. Although acceptable, the statement could be slightly improved by identifying a planned repository or data-sharing platform for transparency (e.g., OSF, Dryad).

5. Is the manuscript presented in an intelligible fashion and written in standard English?

Yes

Comments:
The manuscript is clearly written, well-structured, and follows academic language conventions.

Minor typographical or grammatical issues are negligible. The clarity and professionalism of writing make it easy to follow the study rationale and methodology.

6. Review Comments to the Author

The proposed study is timely and relevant, targeting a key gap in medical simulation pedagogy—whether self-guided debriefing can yield comparable learning outcomes to facilitator-led debriefing in immersive VR environments. The manuscript demonstrates strong methodological rigor, with a thoughtful use of pilot data to inform trial design and robust tools to evaluate both technical and non-technical outcomes.

A. **Comments on methods' section:**
   Strengths:

- The study is methodologically rigorous and adheres to the SPIRIT guidelines.
- It employs a well-defined randomized controlled, single-blinded, non-inferiority design, which is appropriate to answer the stated research questions.
- The use of validated and standardized assessment tools (e.g., CTS, ABCDE checklist, DASH-SV, IMI, NASA-TLX, SUS, VRSQ) strengthens the internal validity and reproducibility.
- Clear blinding procedures for outcome raters and detailed randomization methodology are commendable.
- The intervention and assessment phases are well-illustrated with timelines and scenarios.
- Considerations for VR-induced side effects (cybersickness) and ethical approvals are properly addressed.

Comments for Improvement:

1. **Clarify rationale for non-inferiority margin:**
    - The chosen margin of 0.5 on the CTS scale appears derived from pilot data, but a clearer justification of its clinical relevance is warranted. The margin should reflect what constitutes a non-meaningful difference in teamwork skills.
2. **Stratified randomization detail:**

- Although stratified randomization is mentioned, the strata used (e.g., gender, prior VR/simulation experience) are not clearly specified. This should be detailed to ensure group comparability.

3. **Team composition and matching:**
    - The formation of teams (pairs of students) may introduce variability. Consider clarifying whether any matching criteria (e.g., prior teamwork experience or clinical rotation level) were used to reduce heterogeneity across teams.
4. **Handling missing data:**
    - The statistical plan notes an intention to account for missing data using mixed models, but further detail on handling attrition, particularly in individual-level data (e.g., imputation or exclusion), should be provided.
5. **Blinding limitations:**
    - While rater blinding is described well, the lack of participant blinding may introduce performance bias. Although understandable, it could be useful to acknowledge this limitation more explicitly and discuss mitigation strategies.
6. **Assessment of carryover effect:**
    - As the same individuals are involved in both pre- and post-tests with different scenarios, consideration of potential carryover learning effect or learning due to exposure (rather than intervention) is warranted.
7. **Data safety and monitoring:**
    - The manuscript would benefit from a brief mention of any data monitoring committee or oversight mechanism, particularly in the context of student participants and recorded video data.

B. The Discussion and Limitations Sections:

Strengths:

- The discussion highlights the relevance and potential implications of scalable immersive virtual reality (VR) training, particularly its role in overcoming logistical and cost barriers in simulation-based medical education.
- The manuscript acknowledges the novelty of comparing self-guided versus facilitator-guided debriefing in immersive VR—a relatively unexplored area.

- The potential for broader implementation and democratization of simulation access is well-articulated and compelling.

Comments for Improvement:

1. **Overly Optimistic Tone Without Data:**
   - The discussion leans heavily toward the anticipated benefits of self-guided debriefing without sufficient caution. Given this is a protocol paper, it would be more appropriate to moderate claims and clearly distinguish between expected outcomes and established findings from prior literature or pilot data.
2. **Limited Integration of Pilot Study Findings:**
   - While pilot data are referenced earlier, their implications are not critically analyzed in the discussion. Incorporating a reflection on how these preliminary results informed both the study design and expected impact would enhance the rationale.
3. **Theoretical Underpinnings Need Emphasis:**
   - The discussion would benefit from deeper integration of learning theory—particularly self-regulated learning and cognitive load theory—to better contextualize the study's potential impact and risks (e.g., learner variability, overload).
4. **Scalability Considerations Are Oversimplified:**
   - The promise of wider accessibility is emphasized, but logistical, cultural, and technological barriers to implementation (especially in low-resource settings) should be acknowledged.
5. **Lack of Discussion on Generalizability:**
   - The study uses medical students in a high-income country with access to advanced technology. The discussion should more explicitly address the limitations in generalizing the findings to other populations, healthcare systems, or educational levels.
6. **Limitations Section is Underdeveloped or Implied:**
   - A standalone "Limitations" paragraph or subsection is lacking. The discussion should explicitly list anticipated limitations such as:

- Use of medical students rather than residents or clinicians (may affect ecological validity).
- Short-term assessment only; no long-term follow-up to assess knowledge retention or skill transfer.
- Inability to blind participants, which may introduce bias.
- Potential variability in self-guided debriefing quality despite standardized prompts.

Overall, the manuscript is well-prepared and represents a valuable contribution to the field. Minor revisions as outlined above would strengthen clarity and transparency.

7. Do you want your identity to be public for this peer review?

No

Confidential to Editor

1. Competing Interests:
I have no competing interests.

2. Assistance in Review Preparation:
No assistance was received.

3. Highlighted Submission:
Yes – This protocol is timely and addresses a novel area in medical simulation. It may be of interest to researchers exploring scalable simulation strategies in health professions education.

4. Web of Science Reviewer Recognition:
Yes
