# Supplementary material for: Self-guided versus facilitator-guided debriefing in immersive virtual reality simulation: Protocol for a randomized controlled non-inferiority trial assessing teamwork skills in medical students
Source: PLoS One. 2025 Sep 12;20(9):e0332309. doi: 10.1371/journal.pone.0332309 (PMC12431211; doi:10.1371/journal.pone.0332309)
Supplement: Attachment — Submitted filename: Response to reviewers.docx [file pone.0332309.s006.docx]

**RESPONSE TO EDITOR AND REVIEWERS**

***"Self-guided versus facilitator-guided debriefing in immersive virtual reality simulation: Protocol for a randomized controlled non-inferiority trial assessing teamwork skills in medical students" [PONE-D-25-26268]***

**EDITOR COMMENTS**

1. Please ensure that your manuscript meets PLOS ONE's style requirements, including those for file naming. The PLOS ONE style templates can be found at

https://journals.plos.org/plosone/s/file?id=wjVg/PLOSOne_formatting_sample_main_body.pdf and

[https://journals.plos.org/plosone/s/file?id=ba62/PLOSOne_formatting_sample_title_authors_affiliations.pdf](https://track.editorialmanager.com/CL0/https:%2F%2Fjournals.plos.org%2Fplosone%2Fs%2Ffile%3Fid=ba62%2FPLOSOne_formatting_sample_title_authors_affiliations.pdf/1/010f0198812f8625-6b633265-861a-40c7-9963-a10493b1e7f4-000000/wXiXiEjgHX2rz8HsCKj9zMYAhkQ1eBLyj4yRSBfW8M0=223).

***We thank the Editor for the comment. We have updated the Supporting Information file names to comply with PLOS ONE’s style requirements and have ensured that the rest of the manuscript also adheres to these guidelines.
Please note that the first author’s last name has been changed, as I recently got married and adopted my married name.***

2. Thank you for stating the following in the Acknowledgments Section of your manuscript:

“This work was supported by Gangstedfonden grant number A43424, Helsefonden grant number 21-B-385, and Laerdal Foundation grant number 2024-0321. The funders did not play any role in the study design, data collection and analysis, decision to publish, or preparation of the manuscript.”

We note that you have provided additional information within the Acknowledgements Section that is not currently declared in your Funding Statement. Please note that funding information should not appear in the Acknowledgments section or other areas of your manuscript. We will only publish funding information present in the Funding Statement section of the online submission form.

Please remove any funding-related text from the manuscript and let us know how you would like to update your Funding Statement. Currently, your Funding Statement reads as follows:

“J.K. received a grant for this work from Gangstedfonden, grant number A43424, https://www.gangstedfonden.dk/ and Laerdal Foundation, grant number 2024-0321,https://laerdalfoundation.org/ . A.P. received a grant for this work from Helsefonden, grant number 21-B-385, https://helsefonden.dk/ .

The funders did not play any role in the study design, data collection and analysis, decision to publish, or preparation of the manuscript.”

Please include your amended statements within your cover letter; we will change the online submission form on your behalf.

***We thank the Editor for this comment. We have removed the funding-related text from the manuscript and wish to retain the Funding Statement as currently written.***

3. Please include captions for your Supporting Information files at the end of your manuscript, and update any in-text citations to match accordingly. Please see our Supporting Information guidelines for more information: <http://journals.plos.org/plosone/s/supporting-information>.

***We thank the Editor for this comment and have now included captions for our Supporting Information files at the end our manuscript in line with the above guidelines.***

4. If the reviewer comments include a recommendation to cite specific previously published works, please review and evaluate these publications to determine whether they are relevant and should be cited. There is no requirement to cite these works unless the editor has indicated otherwise.

***Thank you for the comment. The reviewer comments did not include a recommendation to cite specific previously published works.***

5. Please review your reference list to ensure that it is complete and correct. If you have cited papers that have been retracted, please include the rationale for doing so in the manuscript text, or remove these references and replace them with relevant current references. Any changes to the reference list should be mentioned in the rebuttal letter that accompanies your revised manuscript. If you need to cite a retracted article, indicate the article’s retracted status in the References list and also include a citation and full reference for the retraction notice.

***Thank you for the comment. We have reviewed our reference list to ensure it is complete and correct, and did not cite any papers that have been retracted. We have added the following references:***

57.	van Buuren S, Groothuis-Oudshoorn K. mice: Multivariate imputation by chained equations in R. J Stat Softw. 2011;45(3):1–67.

61. Panadero E. A review of self-regulated learning: Six models and four directions for research. Front Psychol. 2017;8(APR):1–28.

62.	Savino S, Mormando G, Saia G, Da Dalt L, Chang TP, Bressan S. SIMPEDVR: using VR in teaching pediatric emergencies to undergraduate students—a pilot study. Eur J Pediatr [Internet]. 2024;183(1):499–502.

**Additional Editor Comments (if provided):**

This study proposal is an interesting and important paper that compares the effects of self-guided compared to facilitator-guided debriefing for immersive virtual reality simulation-based pediatric emergency team training.

It is clear that time and energy have been dedicated to this study and overall, it is a well written study proposal. I congratulate the research team for this excellent work. A few minor comments are given by the reviewers.

***We thank the Editor for this valuable and encouraging feedback on our manuscript. We are grateful for your recognition of the strengths of our study proposal. We have carefully revised the manuscript in accordance with the reviewers’ comments. The specific revisions are described below.
We believe these revisions have enhanced the manuscript’s clarity and overall quality.
We look forward to your further consideration.***

**REVIEWER COMMENTS**

Reviewer's Responses to Questions

**Comments to the Author**

1. Does the manuscript provide a valid rationale for the proposed study, with clearly identified and justified research questions?

**Reviewer #1: Yes**

***We thank the reviewer for confirming that the manuscript provides a valid rationale for the proposed study with clearly identified and justified research questions.***

**Reviewer #2: Yes**

Comments: The manuscript provides a well-articulated rationale grounded in relevant literature for evaluating self-guided versus facilitator-guided debriefing in immersive VR-based simulation. The identified research gap is significant, given the increasing adoption of VR in medical education and the need to assess the pedagogical equivalence of debriefing methods. The research questions are clearly stated, aligned with the study objectives, and justified based on the results of a preceding pilot study.

***We thank the reviewer for the recognition of the significance of the identified research gap, the clarity of the research questions, and their alignment with the study objectives. We are also encouraged by your acknowledgement that the preceding pilot study supports the justification for the present trial.***

**2. Is the protocol technically sound and planned in a manner that will lead to a meaningful outcome and allow testing the stated hypotheses?**

**Reviewer #1: Yes**

***We thank the reviewer for confirming that the protocol is technically sound and well planned.***

**Reviewer #2: Yes**
Comments: The study is designed as a randomized, controlled, non-inferiority trial with appropriate blinding of raters and a robust pretest-posttest structure. The use of validated assessment tools and statistical models (e.g., constrained linear mixed models) supports the technical rigor of the protocol. The inclusion of a power analysis based on pilot data adds strength to the proposed sample size. Exploratory outcomes, assumptions, and blinding are adequately addressed.

***We thank the reviewer for acknowledging our efforts to ensure a robust study design, including the use of validated assessment tools, blinded raters, and power calculation based on pilot data.***

**3. Is the methodology feasible and described in sufficient detail to allow the work to be replicable?**

**Reviewer #1: Yes**

***We thank the reviewer for confirming that the methodology is feasible and described in sufficient detail.***

**Reviewer #2: Yes but**

**Comments:**

**Strengths:**

- The study is methodologically rigorous and adheres to the SPIRIT guidelines.
- It employs a well-defined randomized controlled, single-blinded, non-inferiority design, which is appropriate to answer the stated research questions.
- The use of validated and standardized assessment tools (e.g., CTS, ABCDE checklist, DASH-SV, IMI, NASA-TLX, SUS, VRSQ) strengthens the internal validity and reproducibility.
- Clear blinding procedures for outcome raters and detailed randomization methodology are commendable.
- The intervention and assessment phases are well-illustrated with timelines and scenarios.
- Considerations for VR-induced side effects (cybersickness) and ethical approvals are properly addressed.

**Comments for Improvement:**

- **Clarify rationale for non-inferiority margin:** The chosen margin of 0.5 on the CTS scale appears derived from pilot data, but a clearer justification of its clinical relevance is warranted. The margin should reflect what constitutes a non-meaningful difference in teamwork skills.
- **Stratified randomization detail:** Although stratified randomization is mentioned, the strata used (e.g., gender, prior VR/simulation experience) are not clearly specified. This should be detailed to ensure group comparability.
- **Team composition and matching:** The formation of teams (pairs of students) may introduce variability. Consider clarifying whether any matching criteria (e.g., prior teamwork experience or clinical rotation level) were used to reduce heterogeneity across teams.
- **Handling missing data:** The statistical plan notes an intention to account for missing data using mixed models, but further detail on handling attrition, particularly in individual-level data (e.g., imputation or exclusion), should be provided.
- **Blinding limitations:** While rater blinding is described well, the lack of participant blinding may introduce performance bias. Although understandable, it could be useful to acknowledge this limitation more explicitly and discuss mitigation strategies.
- **Assessment of carryover effect:** As the same individuals are involved in both pre- and post-tests with different scenarios, consideration of potential carryover learning effect or learning due to exposure (rather than intervention) is warranted.
- **Data safety and monitoring**: The manuscript would benefit from a brief mention of any data monitoring committee or oversight mechanism, particularly in the context of student participants and recorded video data.

***We thank the reviewer for this valuable and insightful feedback. Each of the above comments are addressed explicitly below in the section “6. Review Comments to the Author”.***

**4. Have the authors described where all data underlying the findings will be made available when the study is complete?**

**Reviewer #1: Yes**

***We thank the reviewer for confirming that we have described where data underlying the findings will be made available when the study is complete.***

**Reviewer #2: Yes**

Comments: The authors have declared that no datasets were generated at this stage and that deidentified data will be made available upon completion of the study. Although acceptable, the statement could be slightly improved by identifying a planned repository or data-sharing platform for transparency (e.g., OSF, Dryad).

***We thank the reviewer for this suggestion. While a specific data-sharing platform has not yet been selected, we will ensure that deidentified data will be available upon study completion, in accordance with journal and institutional requirements.***

**5. Is the manuscript presented in an intelligible fashion and written in standard English?**

**Reviewer #1: Yes**

***We thank the reviewer for recognizing that the manuscript is presented in an intelligible fashion.***

**Reviewer #2: Yes**
Comments: The manuscript is clearly written, well-structured, and follows academic language conventions. Minor typographical or grammatical issues are negligible. The clarity and professionalism of writing make it easy to follow the study rationale and methodology.

***We appreciate that the reviewer found the manuscript to be clearly written, well-structured, and easy to follow.***

**6. Review Comments to the Author**

***Please note that line numbers in the response to reviewers refer to the “Revised Manuscript with Track Changes” file.***

**Reviewer #1:**

Very minor revision
This is a well written protocol sensibly based on experience from an earlier randomised pilot study. The research questions are clearly specified (lines 153-168). The sample size appears appropriate although the description (lines 307-316) is not entirely clear. Could the authors look at the description again? The analytical methods with respect to the statistical analysis are entirely suitable.

***We thank the reviewer for this encouraging feedback on our manuscript and are pleased that the reviewer found the manuscript to be well written, the research questions clearly specified, and the analytical methods suitable.
We agree that the description of the sample size calculation needed clarification. We have revised the section (lines 311-319) to provide a clearer explanation of the calculation and justification for the non-inferiority margin.***

**Reviewer #2:**

The proposed study is timely and relevant, targeting a key gap in medical simulation pedagogy—whether self-guided debriefing can yield comparable learning outcomes to facilitator-led debriefing in immersive VR environments. The manuscript demonstrates strong methodological rigor, with a thoughtful use of pilot data to inform trial design and robust tools to evaluate both technical and non-technical outcomes.

**A. Comments on methods’ section:
*Strengths:***
• The study is methodologically rigorous and adheres to the SPIRIT guidelines.
• It employs a well-defined randomized controlled, single-blinded, non-inferiority design, which is appropriate to answer the stated research questions.
• The use of validated and standardized assessment tools (e.g., CTS, ABCDE checklist, DASH-SV, IMI, NASA-TLX, SUS, VRSQ) strengthens the internal validity and reproducibility.
• Clear blinding procedures for outcome raters and detailed randomization methodology are commendable.
• The intervention and assessment phases are well-illustrated with timelines and scenarios.
• Considerations for VR-induced side effects (cybersickness) and ethical approvals are properly addressed.

***We thank the reviewer for their thorough and encouraging assessment of our study protocol. We appreciate their recognition of our efforts to ensure methodological rigor, adherence to SPIRIT guidelines, use validated assessment tools, maintain blinding of outcome raters, and address VR-induced side effects.***

***Comments for Improvement:***

**1. Clarify rationale for non-inferiority margin:**
o The chosen margin of 0.5 on the CTS scale appears derived from pilot data, but a clearer justification of its clinical relevance is warranted. The margin should reflect what constitutes a non-meaningful difference in teamwork skills.

***We thank the reviewer for this comment and agree that this part was not sufficiently clarified. We have now clarified the rationale for the chosen non-inferiority margin of 0.5 and why this margin is expected to constitute a non-meaningful difference in teamwork skills (lines 313-317):***

“We set a non-inferiority margin of 0.5 points on the 0-10 CTS© scale, meaning that a difference of less than 0.5 points on the CTS© scale is not expected to constitute a meaningful difference in teamwork skills. The non-inferiority margin was informed by expert consensus, our experience rating teamwork skills in the pilot study, and pilot data (30).”

**2. Stratified randomization detail:**
o Although stratified randomization is mentioned, the strata used (e.g., gender, prior VR/simulation experience) are not clearly specified. This should be detailed to ensure group comparability.

***We thank the reviewer for pointing out this important clarification. Upon revisiting our randomization procedure, we recognize that the term stratified randomization was used in error. In our study, randomization is conducted at the team level, with each team of two students allocated as a unit to either the self-guided or facilitator-guided group at a 1:1 ratio. This constitutes cluster randomization rather than stratified randomization, as no strata (e.g., gender, prior VR/simulation experience) are used in the allocation process. We have corrected the terminology in the Methods section to accurately reflect this (lines 327-331) and addressed the absence of stratification in the “Strengths and limitations” section (lines 441-445):***

“Medical students will enroll in the study in teams of two based on schedule availability, without matching for prior teamwork experience, clinical rotation level, or other characteristics. Each team will be treated as a cluster and randomly allocated to the self-guided or facilitator-guided group at a 1:1 allocation ratio.”

“Fourth, randomization was performed at the team (cluster) level without stratification for potential confounding variables (e.g., prior VR or simulation experience), and team composition was not matched for e.g. prior teamwork experience or clinical rotation level, which may introduce variability in baseline teamwork performance between groups. While baseline characteristics will be compared between groups, chance imbalances cannot be entirely excluded.”

**3. Team composition and matching:**
o The formation of teams (pairs of students) may introduce variability. Consider clarifying whether any matching criteria (e.g., prior teamwork experience or clinical rotation level) were used to reduce heterogeneity across teams.

***We thank the reviewer for this valuable observation. In our study, teams are formed based on schedule availability without matching for prior teamwork experience, clinical rotation level, or other characteristics. We have clarified this in the Methods section (lines 327–331) and added an acknowledgment of the potential heterogeneity this may introduce across teams in the “Strengths and Limitations” section (lines 441–445). See specific wording above under point 2 “Stratified randomization detail”.***

**4. Handling missing data:**
o The statistical plan notes an intention to account for missing data using mixed models, but further detail on handling attrition, particularly in individual-level data (e.g., imputation or exclusion), should be provided.

***We thank the reviewer for highlighting the importance of clarifying our approach to missing data. We have now detailed our plan in the Methods section, specifying that missing outcome data will be handled using linear mixed models, which accommodate data missing at random by including all available data points without requiring imputation. We have also added that for missing individual-level covariate or questionnaire data, we will assess the extent of missingness and apply multiple imputation if missingness exceeds 5% (lines 361-365):***

“Missing data will be handled within the linear mixed model under the assumption that data are missing at random by including all available data points without requiring imputation. For missing individual-level covariate or questionnaire data, we will first assess the extent and pattern of missingness. If missingness exceeds a minimal threshold of >5%, we will apply multiple imputation using chained equations to account for missing values (57).”

**5. Blinding limitations:**
o While rater blinding is described well, the lack of participant blinding may introduce performance bias. Although understandable, it could be useful to acknowledge this limitation more explicitly and discuss mitigation strategies.

***We thank the reviewer for raising this important point. We acknowledge that participant blinding is not feasible due to the nature of the interventions, which may introduce performance bias. We have now explicitly addressed this limitation in the “Strengths and Limitations” section, including a discussion of our mitigation strategies: use of standardized protocols for intervention delivery and performance evaluation, blinding of outcome assessors, validated assessment tools, and randomization to ensure balanced groups (lines 435-440).
Further, we have clarified the limitation and mitigation strategies in the Methods section (lines 336-342):***

“Due to the nature of the interventions, blinding of participants is not feasible. However, participants will not be informed of their group allocation until the intervention day, after completing the pretest, the VR scenarios used for intervention delivery are pre-programmed and identical across the intervention groups to ensure standardization, and the performance evaluation scenarios will follow standardized protocols. The researchers doing the statistical analyses will be blinded to allocation of participants.”

“Third, due to the inherent nature of comparing self-guided with facilitator-guided debriefing, participants cannot be blinded to group allocation, which may introduce performance bias. We mitigate this through standardized scenarios for both intervention delivery and performance evaluation, blinding of outcome assessors, use of validated assessment tools, and randomization to ensure balanced groups.“

**6. Assessment of carryover effect:**
o As the same individuals are involved in both pre- and post-tests with different scenarios, consideration of potential carryover learning effect or learning due to exposure (rather than intervention) is warranted.

***We thank the reviewer for highlighting the importance of considering potential carryover effects due to repeated testing. We acknowledge that using the same participants for pre- and post-intervention assessments could introduce learning effects unrelated to the intervention itself. We have now addressed this explicitly in the Methods section (lines 241-244) and the Strengths and limitations section (lines 446-449), outlining our mitigation strategy of using pre- and post-test scenarios that are distinct but comparable in complexity.
We have also clarified that this bias is expected to be similar across both groups, which may exaggerate the overall intervention effect but should not influence the primary outcome comparing self-guided to facilitator-guided debriefing.***

“To reduce potential carryover or learning effects between pre- and post-intervention assessments, distinct but comparable scenarios are used at each time point. Scenarios are matched in complexity and learning objectives but differ in content to minimize practice effects.”

“Fifth, repeated testing introduces the risk of carryover or practice effects. Although scenarios differ in content but are matched for complexity, repeated exposure may lead to learning independent of the intervention. However, this is likely to affect both groups equally, potentially exaggerating overall improvements but not the primary non-inferiority comparison.”

**7. Data safety and monitoring:**
o The manuscript would benefit from a brief mention of any data monitoring committee or oversight mechanism, particularly in the context of student participants and recorded video data.

***We thank the reviewer for raising the importance of oversight mechanisms, particularly regarding the use of student participants and recorded video data. In accordance with national regulations and institutional policy, this study was deemed exempt from formal review by the institutional research ethics board. Approval for the handling and storage of sensitive personal data, including recorded video material, was obtained from the Danish Data Protection Agency (Privacy: P-2023-167). All data are stored on secure, access-restricted servers, and video files are only accessible to authorized research personnel for assessment purposes. We have added this clarification to the Methods section (lines 305-307) and the Ethics and Dissemination section (lines 383-387).***

*“*Pretest and posttest videos will be stored on a secure hospital drive as logged files, accessible to only to authorized study personnel and are permanently deleted after completion of analyses.”

“Video recordings are stored on secure, access-restricted institutional servers, accessible only to authorized study personnel. All recordings are used solely for blinded performance assessment and are permanently deleted after completion of the analyses in accordance with the data management plan approved by the Danish Data Protection Agency.”

**B. The Discussion and Limitations Sections:
*Strengths:***
• The discussion highlights the relevance and potential implications of scalable immersive virtual reality (VR) training, particularly its role in overcoming logistical and cost barriers in simulation-based medical education.
• The manuscript acknowledges the novelty of comparing self-guided versus facilitator-guided debriefing in immersive VR—a relatively unexplored area.
• The potential for broader implementation and democratization of simulation access is well-articulated and compelling.

***We thank the reviewer for these encouraging comments regarding our Discussion section. We appreciate the recognition of the potential impact of scalable immersive VR training in addressing logistical and cost-related barriers to simulation-based education, as well as the novelty of our comparison between self-guided and facilitator-guided debriefing. We are pleased that our discussion of broader implementation and democratization of simulation access was found to be compelling, and we have aimed to retain and further nuance these aspects in line with the reviewer’s “Comments for Improvement” below, to strengthen the manuscript’s relevance and contribution.***

***Comments for Improvement:***

**1. Overly Optimistic Tone Without Data:**
o The discussion leans heavily toward the anticipated benefits of self-guided debriefing without sufficient caution. Given this is a protocol paper, it would be more appropriate to moderate claims and clearly distinguish between expected outcomes and established findings from prior literature or pilot data.

***We thank the reviewer for the comment and agree that the discussion in the original version leaned toward an overly optimistic tone. We have now revised the tone throughout the Discussion section to clearly distinguish between expected outcomes and established findings, moderating the tone to reflect that this is a protocol paper (see Discussion section, lines 400-422).***

**2. Limited Integration of Pilot Study Findings:**
o While pilot data are referenced earlier, their implications are not critically analyzed in the discussion. Incorporating a reflection on how these preliminary results informed both the study design and expected impact would enhance the rationale.

***We thank the reviewer for this valuable comment. We agree that the discussion would benefit from discussing the implications of pilot study findings. We have now integrated key results from our pilot study, highlighting how these informed the present trial’s design (lines 400-409):***

*“*Findings from our pilot study suggest that both debriefing methods are acceptable, usable, and effective for training interprofessional teams of medical doctors and nurses in a high resource setting (30). We will add to these findings by investigating the two debriefing approaches in a population of more novice learners. In the absence of a facilitator, learners with less developed self-regulated learning skills may struggle to frame their experiences effectively (27,61), miss critical learning points, and experience a high cognitive load (27,28).
Further, our pilot study was based on a convenience sample. This study will add to our pilot findings by applying a non-inferiority RCT design, thus enabling us to investigate whether self-guided debriefing can be considered non-inferior to facilitator-guided debriefing for improving medical students’ teamwork skills.”

**3. Theoretical Underpinnings Need Emphasis:**
o The discussion would benefit from deeper integration of learning theory—particularly self-regulated learning and cognitive load theory—to better contextualize the study’s potential impact and risks (e.g., learner variability, overload).

***We thank the reviewer for this comment and agree that the Discussion would benefit from deeper integration of learning theory. As the reviewer suggested, we have now integrated self-regulated learning and cognitive load theory, to contextualize both the potential benefits and risks of self-guided debriefing (lines 402-405). See specific wording above under point 3 “Limited Integration of Pilot Study Findings”.***

**4. Scalability Considerations Are Oversimplified:**
o The promise of wider accessibility is emphasized, but logistical, cultural, and technological barriers to implementation (especially in low-resource settings) should be acknowledged.

***We appreciate the reviewer’s point and have revised the Discussion to acknowledge potential barriers for wider adoption of VR simulation, including an emphasis on the limited knowledge on the feasibility and effect of VR in low resource settings (lines 412-419).***

**5. Lack of Discussion on Generalizability:**
o The study uses medical students in a high-income country with access to advanced technology. The discussion should more explicitly address the limitations in generalizing the findings to other populations, healthcare systems, or educational levels.

***We agree with the reviewer that the generalizability of our findings is limited by the study population and setting. We have now added a paragraph to the Discussion explicitly highlighting the limited knowledge on the applicability of the study’s findings to other settings, emphasizing the need for future studies investigating the feasibility and effect of VR in settings with limited access to advanced technology, technical support, or prior simulation experience (lines 412-419):***

“However, several barriers to implementing VR-based simulation remain, such as logistical, cultural, and technological barriers (11,30,44,62). Further, this trial involves medical students in a high-income country with access to advanced technology and little is known about the feasibility and effect of VR-based simulation in low resource settings (41,63,64). Future studies could explore the feasibility and effect of VR in other contexts, particularly those with limited access to advanced technology, technical support, or prior simulation experience, to further inform for whom and under what conditions it is most effective.”

**6. Limitations Section is Underdeveloped or Implied:**
o A standalone "Limitations" paragraph or subsection is lacking. The discussion should explicitly list anticipated limitations such as:
♣ Use of medical students rather than residents or clinicians (may affect ecological validity).
♣ Short-term assessment only; no long-term follow-up to assess knowledge retention or skill transfer.
♣ Inability to blind participants, which may introduce bias.
♣ Potential variability in self-guided debriefing quality despite standardized prompts.

***We thank the reviewer for highlighting the need for a more explicit and structured presentation of the study’s limitations. We have now added a standalone “Strengths and Limitations” subsection to the Discussion that directly addresses the points raised, including the use of medical students as participants, the short-term assessment design, the inability to blind participants, and potential variability in self-guided debriefing quality despite standardized prompts, as well as the additional considerations noted in the reviewer’s earlier comments. These changes are reflected in the revised manuscript (full text of the “Strengths and Limitations” section lines 423–453; revisions corresponding to the reviewer’s comments above are inserted below).***

“However, several limitations should be noted. First, the participant sample consists of medical students rather than residents or practicing clinicians, which may limit ecological validity and the direct applicability of findings to clinical practice. Second, the trial evaluates outcomes only in the short term, without long-term follow-up to assess retention of knowledge, maintenance of teamwork skills, or transfer of learning to real clinical environments. However, this design will help ensure a robust dataset with minimal missing data. Third, due to the inherent nature of comparing self-guided with facilitator-guided debriefing, participants cannot be blinded to group allocation, which may introduce performance bias. We mitigate this through standardized scenarios for both intervention delivery and performance evaluation, blinding of outcome assessors, use of validated assessment tools, and randomization to ensure balanced groups.”

“Finally, although self-guided debriefing will be supported by standardized prompts to ensure consistency, there remains a possibility of variability in the depth and quality of reflection achieved between individuals and teams. These factors should be considered when interpreting the results.”

**7. Overall, the manuscript is well-prepared and represents a valuable contribution to the field. Minor revisions as outlined above would strengthen clarity and transparency.**

***We thank the reviewer for the encouraging feedback and constructive suggestions. We have addressed all minor revisions as outlined in the specific comments, revising the manuscript to improve clarity and transparency.***
